# Topics of Public Interest

**Published:** 1913-03

**Authors:** 


### TOPICS OF PUBLIC INTEREST.

PURE FOOD. 196 of 200 women students at a pure food banquet at the University of Chicago, February 6, suffered from ptomaine poisoning. (Note—This is a suspiciously high percentage for bacterial infection and suggests some irritant vegetable cathartic active principle.)

SMALL BABY. Mrs. Paul de Metres of Philadelphia gave birth to twins about the first of the year, weighing respectively 1 and

4½ pounds. The larger one died. The smaller, after 47 days, weighed 1¼ pounds, measured 14½ inches and was apparently healthy.

---

THE CONSOLIDATION is announced of the Medical College of Virginia and the University College of Medicine of Richmond.

---

THE ROCHESTER GENERAL HOSPITAL treated 1015 ward and 1019 private patients in 1912. 247 children were born in the hospital.

---

THE ROCHESTER HOMOEOPATHIC HOSPITAL treated 2128 full-paying patients, 97 part-paying and 536 free patients in 1912.

---

There are TWO PHYSICIANS IN U. S. SENATE: Gallinger of Vermont, a homoeopath who has not practiced for many years and who is considered an anti-vivisectionist and anti-vaccinationist; Harry Lane, newly elected from Oregon, a regular, in active practice up to the present and supposed to favor animal experimentation, vaccination, etc.

---

THE McCLELLAND ANTI-VIVISECTION BILL was killed in N. Y. Senate Committee by a vote of 6-4. Such bills are introduced annually and we repeat our warning that, if the profession continues to oppose investigation and legislation on this subject, a drastic bill may be expected to pass sometime. This does not imply that our policy is that of an anti. On the contrary, we hold that not only institutions but individuals, properly qualified, whether possessing the degree of medicine or not, should be allowed to experiment on animals subject only to such reasonable supervision and restriction as is applied to other matters on which public opinion demands legislation.

---

DR. DANIEL G. CAREY of Elmira was indicted for manslaughter January 29, death being due to an abortion. Dr. Carey is not listed in the State Directory but is in Polk's Directory, having graduated from the Eclectic Medical College of Pennsylvania, in Philadelphia, in 1870. This school has been extinct since 1880.

---

U. S. PUBLIC HEALTH SERVICE: .EXAMINATION OF CANDIDATES FOR ASSISTANT SURGEON. Examinations will be held in Washington, April 7. Application for blanks should be made immediately to the Surgeon-General. The initial salary is $2,000, with quarters, successive promotions increasing the salary to the equivalent of $6,000 toward the close of one's life. This is, in our opinion, the most pleasant and most desirable from the standpoint of professional ambition, of all the government services.

---

TORONTO had 130 cases of typhoid with 25 deaths in 1902; in 1903, 156 cases, 35 deaths; in 1904, 133 cases, 41 deaths; in 1905, 197 cases, 45 deaths; in 1906, 259 cases, 63 deaths; in 1907, 186 cases, 58 deaths; in 1908, 201 cases, 60 deaths; in 1909, 331 cases, 77 deaths; in 1910, 739 cases, 151 deaths; in 1911, 520 cases, 81 deaths; to December 26th in 1912, 276 cases, 54 deaths. Note—As the average mortality of typhoid is about 10 per cent, it is evident that many non-fatal cases were not reported.

---

SMALLPOX IN QUEBEC. To December 20th, 1912, there were 971 cases of small pox in the Province of Quebec for the six months as follows: July, 106 cases; August, 103; September, 63; October, 119; November, 495; December, 84.

According to Dr. Elzear Pelletier, the Secretary of the Quebec Board of Health, these figures are only a small percentage of the cases, as many municipalities refuse to report their cases of contagious diseases.

---

TUBERCULOSIS AND TELEPHONES. *Dominion Medical Monthly*, January, 1913. So far as is definitely known the tubercle bacillus has been recovered from a telephone swab but once. Prof. Klein found this in 1908 after examining a number of transmitters. In 1905 he had undertaken to conduct these investigations, but failed to find either the tuberculosis or diphtheria bacillus. Spirta recently confirmed these investigations, and although his experiments extended over a year, he did not recover the tubercle bacillus in one instance. Some instruments used were in a hospital for consumptives by patients actually suffering from the disease. Spirta concludes "that the transmission of tuberculosis through the medium of the telephone mouthpiece is practically impossible."

---

THE DEROALDES PRIZE. A gold medal is offered by the American Laryngological Association for the best essay upon some subject relating to laryngology and rhinology by any practitioner in regular standing in the United States or Canada. The essays must be typewritten in English and be accompanied by a sealed envelope containing the author's name and the title of the paper. They must be sent to the secretary, Dr. Harmon Smith, 44 West Forty-ninth street, New York, before May 1, 1913.

---

STREET AREA. For an average American city, with ordinary streets 50-60 feet wide (average for Buffalo 55), a few main thoroughfares 100 feet (99 for Main street, 100 for Richmond and Porter) and parkways of 200 feet with double driveways and grass plots, 20-30 per cent. of the total area is devoted to highways. The newer cities, with ideally wide streets may devote 40 per cent. to this purpose.

---

NOTIFICATION OF VENEREAL DISEASE. The N. Y. City Health Department has ordered superintendents of institutions and has requested private practitioners to give notification of all cases of venereal disease with the source of infection, if possible. The Medical Association of Greater New York City has adopted resolutions condemning this action, the assertion being made that the Health Department contemplates the establishment of free dispensaries for treating cases and has been prevented only by the failure to secure an appropriation of $50,000. The opposition is based both on the possible infringement on private practice and on the general principle of privileged communications by patients.

---

DISCONTINUANCE OF FREE ANTITOXIN. The N. Y. City Health Department has discontinued the practice of supplying free antitoxin for diphtheria, after eighteen years of this policy. The general adoption of this method is given as the reason. Provision will still be made for indigent cases.

---

ASSISTANT IN EXPERIMENTAL THERAPEUTICS, Phillippine Service, salary $2,000. Applicants must be males, with the degree of M. D. and must have had at least one year's experience in post graduate research work or its equivalent. The further requirement that they must have reached their eighteenth year seems superfluous. Choice is made on examination of credentials submitted. Apply to U. S. Civil Service Commission, Washington, D. C., for Form B. I. A. 2, before March 10. (Note—It strikes us that selection by examination of credentials, with public notice sent to monthly journals which appear about five days, on the average, before the papers must be at the office of the Civil Service Commission, is something like Hamlet with Hamlet left out.)

---

MEASLES IN JAMESTOWN, N. Y., 1100 cases were reported up to February 14, with seven deaths. It is worth remembering that while the direct proportionate mortality of measles is not very high, the marked prevalence of the disease and the predisposition which it leaves for tuberculosis, renders it one of the most important unsolved problems in medicine.

PUMPAGE OF WATER IN THE TONAWANDAS. Tonawanda pumped over five million gallons of water a day during January, there being only one fire. 143 tons of coal were used. North Tonawanda pumped nearly 169 million gallons for the same month. Nearly 8 millions a day were pumped during the very cold weather and, at the time of the fire in the Goundry Street School, the pressure could not be raised above fifty pounds. The problem of water supply to keep pipes from freezing is a serious one. Yet, we hold that it is not only human nature but sound economy to allow the waste (?) of a few cents worth of water to prevent a plumbing bill of several dollars and a general insistence on non-freezing plumbing—if such a thing is possible at all—must mean a great increase in the cost of living, both on account of the additional capital invested in plumbing and general construction of houses, and on account of fuel. We do not see how the rent—or the up-keep of a privately owned residence—can be kept within five dollars a month of its present average or how cheap, separate homes with yards can be maintained against the gradual encroachment of apartment buildings, unless water departments generally recognize the incontestable fact that still water will freeze in pipes when the temperature falls below zero centigrade; and the principle of economics that it is no more waste to use a few gallons of water to save two hundred dollars' worth of plumbing, than a quart to wash a penny tumbler.

---

CENTENNARIANS.—The following cases of persons who have reached ages about 100 have been gathered from various sources:

Mrs. Mary Stockham, died October 1, 1912, at Stogurzy, Eng., born August 24, 1812.

Lucy Frey, negress, born a slave June 20, 1790, is still at work as a laundress in Culpepper County, Va.

Mrs. Mary Ann Foley of Denver, born in Ireland 111 years ago, is surprised at being sick for the first time in her life.

Mrs. Elizabeth Thompson, negress, died early in December, 1912, in Patterson, N. J., said to be 115 years old. Her husband lived to be 109. Twenty-one of her twenty-two children and fifty grandchildren survive.

Carsamine Paige, said to have been the oldest newsboy in the world, died at Joliet, Ill., January 2, 1913, aged 105.

Mrs. Maggie Adams, an Indian, died recently at Garibaldi, near Bay City, Ore., at the reputed age of 113.

Joseph Harshi, a carpenter, born in Germany in 1802, came to America at the age of 40, ceased using alcoholic beverages at 80 and tobacco at 105. He died in Chicago, October 21, 1912, aged 110. He is survived by five sons, seventeen grandchildren and five great-grandchildren.

Garret Abers, of Salamanca, was born April 3, 1813, served through the Civil War and is still in good health.

Mrs. Mary Besant, born August 8, 1811, died at Clevedon, Somersetshire, Eng., November 25, 1912.

Charles Cauley, born in Ireland in 1803, died December 25, 1912, at Copper, Tex., and was engaged in active work as a farmer until two months previously.

Mrs. Mary Nideau, born April, 1808, thrice married, died December 19, 1912, at Hampden, Me. She was in vigorous health till shortly before death. She had no living descendents.

A Georgia man, 106 years old, has been sentenced to the chain gang. Anthropologists will regard the incident as corroborative of the adage that the good die young.

Mrs. Mary Ann Peterson, born in New London, Conn., August 22, 1807, died in the same place December 29, 1912.

Mrs. Currance Gould, widow of Dr. Wm. Gould, died in Buffalo January 26, 1913, aged 97.

Wm. J. Ray, one week over 107 years old, was fined for his sixth offense in selling liquor illegally, in Hartford, Conn., January 27, 1913.

Mrs. Samantha Nellis of Naples, N. Y., celebrated her 103d birthday in January, 1913. She is still in good health, though slightly deaf. Her father, Elijah Stanton, was born at Preston, Conn., in 1754, and served in the Revolution.

Mrs. Ann Lacy of Ramsgate, Eng., born December 29, 1812, received congratulations from the king on her hundredth birthday.

M. T. Kreppa, said to be the oldest Odd Fellow in the world, died at Washington, January 30, 1913, aged 95.

Mrs. Marcelina Elisalda, a widow, aged 105, recently married a man of 80 at Los Angeles, but died a few days later.

Mrs. Mary Moore Merrill, Jamestown's (N. Y.) oldest resident, celebrated her 98th birthday January 31, 1913. She is in good health and active.

Mrs. Daniel Swinford, who died on January 3, at Minster, Isle of Thanet, England, was locally reputed to be 100 years of age.

Mrs. Moore of Plymouth, Devonshire, is said to have been born on January 20, 1811.

Mary Fehily, who died on January 5 at Middleton, East Cork, Ireland, was alleged to be 104 years old. She had for many years practised the habit of smoking tobacco.

Wah-Hah-Gun-Ta, a Blackfoot Indian chief at the Glacier Reservation, who is known to have visited Thomas Jefferson while the latter was president, is reported to have been born in 1781. He is said to preserve still an active and vigorous physical health.

James Thomas, who died last week at Monett, Mo., is said to have been born in 1807.

Sarah Ann Byles, a negress, who died on January 29 at Frederick, Md., was locally reputed to have been born in 1798.

---

LACK OF APPRECIATION OF FRANCHISE. About fourteen million votes were cast at the latest presidential election. Approximately 30 per cent. of a total population should normally consist of males over 21. With allowance for increase in population for the two years since the latest census, there were about twenty-eight million males of voting age and about fourteen hundred thousand women of voting age in the six states having the franchise. In other words, the presidential election which normally calls out the highest vote, was participated in by less than half of those therotically eligible. About three million (nearly half of the foreign born) were not naturalized and allowance must be made for sickness, absence, removal within the varying period of residence required by different states and election districts, conviction of crime and other causes. But, at the best, there is a surprisingly large number of men who do not appreciate the franchise and this implies a sociologic problem close enough to professional interests to warrant its being mentioned here. The following statistics which must, on the average be increased by nearly 4 per cent. to correspond to the natural increase of population between June, 1910, and November, 1912, are furnished by the courtesy of the Bureau of the Census:

MALES OF VOTING AGE, 1910.

UNITED STATES, EXCLUSIVE OF OUTLYING POSSESSIONS.

TOTAL, 26,999,151.

| | |
|---|---|
| Native white— | |
| Native parentage | 13,211,731 |
| Foreign or mixed parentage | 4,498,966 |
| Foreign-born white | 6,646,606 |
| Naturalized | 3,035,333 |
| First papers taken out | 570,588 |
| Alien | 2,265,121 |
| Unknown | 775,564 |
| Negro | 2,459,327 |
| *All others | 182,521 |

FEMALES OF VOTING AGE.

| NATIVE WHITE. | Tl. for 6 States |
|---|---|
| Total females 21 and over | 1,346,925 |
| Native Parentage | 654,784 |
| Foreign or mixed parentage | 333,925 |
| Foreign born white | 327,682 |
| Negro | 13,488 |
| *All others | 17,046 |

Six States—California, Colorado, Idaho, Utah, Washington and Wyoming.

*Includes Indians, and Chinese, Japanese and other Asiatics.

35TH ANNUAL REPORT OF THE CHARITY ORGANIZATION SOCIETY OF BUFFALO. The city out-door relief has decreased from $112,053 in 1876 to $43,860. 1.5 per cent. of the population is aided by the Society and the Poor Master instead of 10 per cent. Only about a third of applicants for assistance require financial aid, the rest need legal advice, institutional care, medical treatment and guidance. Lack of employment is a minor factor. 80 per cent. of the aid is due to partial or complete disability and sickness and, of the sickness, only about a third is due to lust and intemperance. About twenty years ago the reviewer was in rather close touch with certain phases of relief work, and, at a rough estimate, two-thirds of the poverty was due to shiftlessness, comparatively little to alcohol. Almost never was a really deserving case encountered, except in the sense that women and children might require aid on account of the delinquency of the nominal supporter of the family. Not to be unfair to the men, it should also be stated that many cases of acute poverty were the fault of wives and children. It is only fair to state that institutional charity, philanthropic organizations, etc., which are supported on a magnificent scale of generosity as compared with conditions thirty-five years ago, account for a large share of the apparant diminution of charity. Thanks to the late J. N. Adam, the Society will soon move into new quarters at 181-185 Franklin street, and will have room for various other philanthropic societies. Not only will the new quarters expedite the good work of the Society, but, as the site is rapidly increasing in value for business, it is probable that, within a few years, the Society can again move to equally convenient quarters and with a considerable capital.

---

DOCTOR WANTED—At Viola, Delaware. There is a good opening for a physician in this town. Inquire of Henry De Blois.

